# Components of Empowerment Among Family Caregivers of Community-Dwelling People With Dementia in Japan: A Qualitative Research Study

**DOI:** 10.1097/JNR.0000000000000430

**Published:** 2021-04-09

**Authors:** Sayori SAKANASHI, Kimie FUJITA, Rie KONNO

**Affiliations:** 1 PhD, RN, Senior Assistant Professor, Faculty of Medicine, School of Nursing, Fukuoka University, Fukuoka, Japan; 2 PhD, RN, Professor, Faculty of Medical Sciences, Department of Health Sciences, Kyushu University, Fukuoka, Japan; 3 PhD, RN, Professor, School of Nursing, Hyogo University of Health Sciences, Hyogo, Japan.

**Keywords:** dementia, empowerment, family caregiver, Japan, qualitative research

## Abstract

**Background:**

Family caregivers of people with dementia (PWDs) experience significant physical, psychological, and social burdens. Empowerment, which refers to the process of gaining power in society through behavioral change, is important to coping successfully with care-related burdens. The high burden of care faced by family caregivers in Japan often makes accepting social support difficult for caregivers of PWDs, resulting in feelings of isolation. Clarifying what components constitute empowering experiences for family caregivers of PWDs is necessary to gain a better understanding of their empowerment experiences and to develop relevant support schemes.

**Purpose:**

This study was developed to describe the components of empowerment experienced by family caregivers of community-dwelling PWDs in Japan.

**Methods:**

This qualitative descriptive study used semistructured in-depth interviews to explore components of empowerment experienced by family caregivers of adults/older adults with dementia. Purposive sampling was used to recruit 20 family caregivers (age range: 50–87 years) from four self-help groups. A qualitative content analysis method was used to analyze the data. The components derived from the interviews were reviewed by three nursing researchers not directly involved in this study who are specialists in qualitative research and geriatric nursing.

**Results:**

Four categories and 12 subcategories were derived to illuminate the components of empowerment among family caregivers of PWDs. Specifically, these categories were as follows: (a) proactive aspects of dementia care that were acquired through the caregiving experience, (b) creating a relationship that respects PWDs, (c) Building relationships based on mutual understanding of one's surroundings, and (d) understanding the social aspects of dementia care.

**Conclusions/Implications for Practice:**

The findings of this study provide additional understanding of the components of the empowerment experiences of family caregivers of PWDs in Japan and in other East Asian countries experiencing increasing dementia diagnoses and population aging. In addition, the structural components of empowerment offer a useful perspective for health professionals on assessment and intervention that is framed on the cultural characteristics of East Asia. Ultimately, the results suggest that healthcare professionals should develop intervention programs that are tailored to the needs of caregivers at different levels of empowerment.

## Introduction

An estimated 50 million people worldwide currently have dementia, and this number is expected to increase through the coming years (Alzheimer's Disease International, 2020). Dementia affects memory, cognition, behavior, and emotion and increases difficulties with daily living. It has been reported that family caregivers of people with dementia (PWDs) face greater chaotic situational changes, conflictual emotional adaptations, and multiple functional losses than caregivers of persons with other diseases (Che et al., 2006). Therefore, it is critical to develop support for family caregivers of PWDs to maintain and improve their quality of life, particularly because they experience significant physical and psychosocial burdens.

Numerous studies have examined depression and the burden of caregiving in family caregivers of PWDs, with interventions (e.g., education and counseling) aimed at reducing these burdens (Blom et al., 2015). However, in addition to addressing negative aspects, it is also important to focus on the positive aspects that family caregivers gain through caregiving, with the purpose of developing relevant and effective support strategies for family caregivers of PWDs.

The positive concept of empowerment refers to the improved status, changed behaviors, and increased power in society of people who are socially labeled or otherwise facing discrimination (Dooher & Byrt, 2012). Prior research indicates that empowering family caregivers is effective in reducing their physical and mental burdens, increasing self-efficacy, acquiring ongoing social support (Perlick et al., 2013), and developing further mutual trust with the care receiver and their families, as well as for growing as people through their experiences (Jones et al., 2011). Alzheimer's Disease International (2019) highlighted the problem of dementia-related stigma, reporting that over 35% of caregivers worldwide have hidden the diagnosis of dementia. Consequently, this organization has a clear mission to empower people living with dementia. Increasing empowerment in family caregivers of PWDs who have experienced physical and social burdens is crucial to restoring their position and maintaining/improving their quality of life.

Japan has the highest aging rate worldwide, and the population of PWDs in Japan is increasing rapidly. The Japanese Long-Term Insurance System was initiated in 2000 (Iwagami & Tamiya, 2019) to provide assistance to people with care needs. However, this system is not sufficient, as its use is limited by the minimum eligibility threshold set for care provision. In addition, Japanese families traditionally tend to fulfill caregiving responsibilities when older adult family members require care (Kumagai & Ishii-Kuntz, 2016). In other words, family caregivers may have difficulty accepting social support, even when available, resulting in social isolation and a poorer quality of life (Montgomery et al., 2018). In the Japanese cultural context, family caregivers experience significant social pressures related to family caregiving (Kumagai & Ishii-Kuntz, 2016). Kitamura et al. (2019) reported that Japanese family caregivers of PWDs experience considerable isolation and anguish after learning of the diagnosis. Therefore, it is particularly important that Japanese family caregivers who are physically, psychologically, and socially vulnerable acquire knowledge and information, accept support, and develop empowerment proactively.

Empowerment is a multidimensional concept (Gibson, 1991). A literature review reported that, from the perspective of healthcare service users, empowerment comprises cognitive, emotional, and behavioral components (Dooher & Byrt, 2012). Several concept analysis studies in the literature address the components of empowerment in the context of service users (Gibson, 1991; Rodwell, 1996; Sakanashi & Fujita, 2017; Wåhlin, 2016).

According to one analysis study, empowerment among family caregivers of adults and older adults is a comprehensive concept that incorporates the qualities of coping, self-efficacy, autonomy, and self-determination (Sakanashi & Fujita, 2017). Only one previous study in Taiwan has described the components of empowerment for family caregivers of PWDs (Che et al., 2006). However, in that study, all of the participants were women, whereas more than 30% of Japanese family caregivers are men. Notably, it has been reported in Japan that the coping strategies used by male caregivers differ from those used by female caregivers (Nishio & Ono, 2015). Therefore, as the concept of coping is a component of empowerment (Sakanashi & Fujita, 2017), the available literature pertaining to empowerment may not reflect the characteristics of empowerment among family caregivers of PWDs in Japan. Although the focus of Che et al. (2006) is on support within the family, the empowerment of family caregivers also requires support from their surroundings (Ducharme et al., 2002). In addition, few studies have reported on qualitative data related to empowerment among family caregivers of PWDs (Nomura et al., 2009), with available studies focusing on interventions and collected data representing assessments of those interventions only. Empowerment among family caregivers of PWDs has not been studied comprehensively to date. Thus, clarifying empowerment among Japanese family caregivers of PWDs and developing support strategies to increase their empowerment are critical, especially in light of Japan's super-aging society.

The aim of this study was to describe the components of empowerment among family caregivers of community-dwelling PWDs in Japan based on the experiences of family caregivers. Following Sakanashi and Fujita (2017), empowerment among family caregivers of PWDs is defined in this study as

*Positive control of one's mind and body, cultivating a positive attitude, proactively attempting to understand one's role as a caregiver to improve caregiving capabilities, focusing on others as well as oneself, supporting the independence of the care-recipient, and creating constructive relationships with other people surrounding them. (p. 6)*

## Methods

### Study Design

A qualitative descriptive design (Sandelowski, 2000) was used to gain a rich understanding of the components of empowerment among family caregivers of PWDs.

### Population and Sample

Family caregivers of PWDs were recruited from four self-help groups in Japan. Inclusion criteria were at least 20 years old and fluent in spoken Japanese. Purposive sampling (Sandelowski, 2000) was used to recruit a mix of caregivers (i.e., male, female, husbands, wives, sons, and daughters), caregiving durations, and the types of dementia diagnoses to promote sample representativeness across study sites. Long-term participation in the field and assiduous observation yield reliable information (Flick, 2011). Thus, before conducting the interviews, the authors participated in the four self-help groups over a 4-year period to develop relationships with potential participants. Subsequently, those family caregivers of PWDs who had talked to one of the group administrators about the empowerment of family caregivers were targeted for recruitment as study participants.

### Data Collection

A semistructured interview method was used to collect data between October 2015 and July 2019. Detailed, in-depth interviews were conducted using an interview guide based on the empowerment of family caregivers (Sakanashi & Fujita, 2017). Interview questions were as follows: “Tell me about your physical and mental condition and how you have controlled it,” “Tell me about your feelings and thoughts about your caregiving role,” “Explain what you have done to improve your caregiving skills and knowledge,” “Describe how you have supported yourself and others,” and “Explain how you have worked to build relationships with other people and those surrounding them.” We collected information on family caregivers' demographic characteristics, including gender, age, employment status during caregiving, relationship to the PWD, family cohabitation status, PWD's dementia type, duration of caregiving, and level of caregiving required.

In terms of procedure, representatives of the self-help groups were first informed of the purpose of this study. Next, the researchers explained the research purpose and procedures to recruited participants. After obtaining written and informed consent, the one-on-one, semistructured interviews were conducted in an environment that was comfortable and safe, encouraging participants to engage in the interview openly and freely. Interview sessions were recorded using a digital voice recorder with the consent of participants. Participants' expressions and movements during interviews were also documented in field notes.

### Analysis

As mentioned previously, data were analyzed using qualitative content analysis (Elo & Kyngäs, 2008) after the interviews had been transcribed verbatim. In the first phase, the first author led the analysis after becoming familiar with the data. All of the interview transcripts were read repeatedly, and as many codes as necessary were written down to describe all aspects of the content. At this stage, the codes were identified on coding sheets and categories were generated. After this process of open coding, lists of categories were grouped under higher-order codes. Each category was named using content-characteristic words, and subcategories with similar events and incidents were grouped together. The second phase involved another researcher reading the transcripts and verifying the data categorization. In the third phase, the results of analyses were discussed and the components were developed. Peer checking by three nursing researchers with specialist knowledge in qualitative research and geriatric nursing who were not otherwise involved in this study was performed to ensure rigor.

### Ethical Considerations

This study was approved by the ethical review board of Fukuoka University, Japan (ID Number 439). Participants were informed of the study purpose and procedures, the potential for publication, and the confidentiality of their data as well as their right to refuse. Written and informed consent was obtained from all of the participants before the interviews took place.

## Results

### Participant Characteristics

Twenty family caregivers participated in the interviews. Participant characteristics are shown in Table 1. The mean age of participants was 69.2 years (range = 50–87 years), 10 participants were men, 10 were retired, 12 were the spouse of the care recipient, and nine reported a caregiving duration of 5–10 years. Nine of the care recipients required extensive caregiving, and 10 had Alzheimer's disease.

**Table 1.** Demographic Characteristics of the Participants (*N* = 20)

Characteristic	*n*	%
Gender		
Male	10	50
Female	10	50
Age (years; *M* and *SD*)	69.2	9.5
Range	50–87	
Employment status during caregiving		
Full-time worker	3	15
Part-time worker	1	5
Self-employed	2	10
Full-time homemaker	4	20
Retired	10	50
Relationship to the person with dementia ^a^		
Spouse	12	60
Son	3	15
Daughter	5	25
Sister	1	5
Family members living together		
Yes	10	50
No	10	50
Duration of caregiving ^a^		
< 1 year	2	10
1–5 years	7	33
6–10 years	8	38
> 10 years	4	19
Interview length (minutes; *M* and *SD*)	95.3	34.8
Range	47–180	
Caregiving required		
Mild	4	20
Moderate	7	35
Extensive	9	45
Type of dementia ^a^		
Alzheimer's disease	10	50
Dementia with Lewy bodies	4	20
Frontotemporal dementia	4	20
Vascular dementia	3	15

^a^ Includes duplication because one family member in this study provided care to two people with dementia (a mother and a sister).

### Interviews

Interviews with the family caregivers lasted 47–180 minutes (*M* = 95.3 minutes) each.

### Description of Categories

The extracted analysis components are presented in Table 2. The components of empowerment experienced by family caregivers of PWDs extracted in this study were captured by four categories and 12 subcategories. Specific examples from participants' responses are included below to illustrate each subcategory. Case identifiers are denoted by “[Mr./Ms., family caregiver].”

**Table 2.** Components of Empowerment Among Family Caregivers of Community-Dwelling People With Dementia

Category (*n* = 4)	Subcategory (*n* = 12)
Proactive aspects of dementia care that were acquired through the caregiving experience	1. Becoming accepting of one's own caregiving role 2. Acquiring caregiving-related coping strategies 3. Improving one's sense of self-efficacy about caregiving 4. Changing ways of thinking and attitudes toward dementia care 5. Learning to take care of one's own health and quality of life while caregiving
Creating a relationship that respects people with dementia	1. Maintaining an affectionate relationship with the person with dementia 2. Understanding the feelings of the person with dementia
Building relationships based on mutual understanding of one's surroundings	1. Developing mutual cooperation with other family members 2. Constructing collaborative relationships with people around them 3. Having peers who share caregiving experiences
Understanding the social aspects of dementia care	1. Becoming aware of the social system that surrounds dementia 2. Actively sharing one's own caregiving experiences with newer caregivers

#### Proactive aspects of dementia care that were acquired through the caregiving experience

This category reflects how family caregivers perceive their role as caregivers and how they perform their caregiving responsibilities well by gaining coping skills or improving self-efficacy. The participants were devoted to caregiving and had also begun paying greater attention to their own life and health, which had a positive impact on care.

(1) Becoming accepting of one's own caregiving role: On the basis of this subcategory, the participants were prepared to take on their caring role.

*When I decided to take care of my mother at home, I thought that there was no one else to take care of her. I thought I should not let my wife take care of my mother, because my wife had cared for her parents in the past. At that time, she was very tired of caregiving. So, I separated from my wife and started living with my mother to provide care*. [Mr. D, family caregiver]

(2) Acquiring caregiving-related coping strategies: In this subcategory, the participants adopted methods of coping with their caregiving responsibilities:

*Until now, my relationship with my father has not been good. So at first, I couldn't take care of him kindly. However, I made an effort to think of him not as my father, but as a stranger. Then it became possible to take care of him objectively. This method seems good for me. I feel comfortable and can take care of him*. [Ms. N., family caregiver]

(3) Improving one's sense of self-efficacy about caregiving: In increasing their care experience, the participants gained confidence:

*Twenty years ago, my mother had dementia. I worked hard to provide care at home. I think I had acquired my own way of caring. It is also hard for me to take care of my older sister this time, but I think I can cope [with] it better than before*. [Ms. P., family caregiver]

(4) Changing ways of thinking and attitudes toward dementia care: For this subcategory, family caregivers had changed over the course of providing care. For example, Mr. K. reflected on those who took time to build relationships with him and explained that his way of thinking about dementia care has changed:

*Dementia is not difficult. It is important that caregivers understand and agree with it. Now that I understand dementia, I can enjoy it with her every day. If I could have understood more, I would not have worried so much about my wife having dementia. I didn't know so I regretted my wife's dementia many times*. [Mr. K., family caregiver]

(5) Learning to take care of one's own health and quality of life while caregiving: The participants were positively influenced by other family caregivers of PWDs and began to take better care of themselves:

*I had no interest in my health until I took care of my wife. However, because only I can take care of her, I cannot get sick now. In particular, I do not want to get dementia. That's why I stopped drinking and I try to live a healthy life as much as possible. I received advice from other family caregivers of PWD*. [Mr. I., family caregiver]

#### Creating a relationship that respects people with dementia

This category reflects how caregivers respect and take care of their care recipient.

(1) Maintaining an affectionate relationship with the PWD: This subcategory describes the good relationships forged by the participants with their care recipients. For example:

*My wife married me when she was younger. She is 10 years older than me. I have been extremely grateful to her for a long time, and I have a strong desire to support her for the rest of my life. So, I decided to live with her for the rest of my life*. [Mr. K., family caregiver]

(2) Understanding the feelings of the PWD: In this subcategory, the participants expressed the belief that PWDs should be treated like normal people:

*Yesterday, my father complained to the manager that the day service was boring. I was surprised that he was right. I found that my father had a reason not to go to the day service. I used to think that people with dementia were not thinking about anything. So, I must understand my father's wish not to attend*. [Mr. M., family caregiver]

#### Building relationships based on mutual understanding of one's surroundings

This category reflects how family caregivers build relationships with others around them. The participants expressed a high degree of trust in other family caregivers of PWDs.

(1) Developing mutual cooperation with other family members: The participants fostered good relationships with other family members through receiving physical and psychological support from them:

*When my mother left [the] house alone, my son searched for her with me. My husband was often not at home [because he was] at work, so I was really saved. My family accepted my staying home to provide care and helped me so I could provide care [to her]*. [Ms. B., family caregiver]

(2) Constructing collaborative relationships with people around them: The participants built relationships with others with whom they could consult and from whom they could get help. For example:

*I told my neighbors openly that my husband had dementia, so they were very kind to us. In particular, my neighbor always took my husband and us together for a drive once a month.* [Mrs. E. family caregiver]

(3) Having peers who share caregiving experiences: The participants emphasized their relationships with caregivers of people with the same type of dementia:

*I went to many self-help groups. When my wife and I took part in a self-help group, there were about 30 women who had experienced [caregiving]. They also took care of my wife as well as me. I trust them [the] most*. [Mr. G., family caregiver]

#### Understanding the social aspects of dementia care

This category reflects the understanding of family caregivers about how dementia is recognized in wider society and in their community. This understanding develops through experiences with caregiving and support received from people around them. This support and their experiences influenced the behavior and attitudes of the participants.

(1) Becoming aware of the social system that surrounds dementia: Mrs. F., who had encountered a good supporter, noticed a social problem surrounding dementia care and stated her opinion regarding it:

*I think if there are services that we can feel free to access to take care of people with dementia, it would make it easier for family caregivers. If we could spend more free time alone, away from with people with dementia, we could lighten our burden and maintain good relationships with people with dementia. Such services are not enough. I wish I could be able to [perform] caregiving more easily. I often talk about this with other family caregivers of PWD*. [Ms. F., family caregiver]

(2) Actively sharing one's own caregiving experiences with newer caregivers: Some of the participants wanted to support other PWDs and their families going forward. For example:

*It is really hard to take care of [someone with dementia] for the first time. So, I hope that I can share my care experience with them [newer caregivers]. Now, I am working as a supporter in a self-help group*. [Mr. J., family caregiver]

## Discussion

In this study, the components of empowerment experienced by family caregivers of community-dwelling PWDs in Japan were examined. The components of empowerment were described using four categories: (a) proactive aspects of dementia care that were acquired through the caregiving experience, (b) creating a relationship that respects PWDs, (c) building relationships based on mutual understanding of one's surroundings, and (d) understanding the social aspects of dementia care.

The category “proactive aspects of dementia care that were acquired through the caregiving experience” reflected how the participants proactively improved their coping skills and self-efficacy to better provide care. This category includes four of the six concepts proposed in a previous concept analysis that examined empowerment among family caregivers (Sakanashi & Fujita, 2017). These four overlapping concepts are “proactive caregiving,” “improvement in caregiving capabilities,” “cultivation of positive feelings,” and “positive control of mind and body.” Che et al. (2006) reported that the process of empowering family caregivers of PWDs includes “care abilities,” “emotional reconstruction,” and “life management.” These aspects are similar to those represented in the category in this study: “proactive aspects of dementia care that were acquired through the caregiving experience.” In a previous study, self-determination in family caregivers was shown to be an important motivation for care and necessary for empowerment (Wu, 2008). In addition, caregivers who are empowered tend to proactively acquire useful information about healthcare, support, and legal procedures (Sakanashi & Fujita, 2020), whereas empowered family caregivers learn to control their mind and body by acquiring coping strategies (Chiu et al., 2013) and seeking support (Polgar, 2009).

In this study, the category “creating a relationship that respects PWDs” reflects how the participants respected and looked after their care recipients. This category is similar to “support for the independence of the care-receiver,” one of the six concepts proposed in the concept analysis of Sakanashi and Fujita (2017). Nomura et al. (2009) reported that family caregivers must acknowledge the potential for PWDs to become empowered. Empowered caregivers believe that care recipients should solve problems independently and that, instead of doing everything for PWDs, they should promote care-recipient independence by giving them a new role and by creating opportunities for interacting with others (Nomura et al., 2009).

The category “building relationships based on mutual understanding of one's surroundings” reflects how the participants built relationships with others in their surroundings. This category is similar to the “constructive relationships with people surrounding them” concept proposed by Sakanashi and Fujita (2017). In prior studies, empowered caregivers have reported receiving support from others (Ducharme et al., 2002), sharing similar feelings (Shawler, 2006), and carrying out activities together (Che et al., 2006) and, as a result, participated in social activities.

The category “understanding the social aspects of dementia care” reflects the understanding of the participants regarding how dementia is recognized in society and in their communities. As noted by Alzheimer's Disease International (2019), stigma-related problems affect PWDs and their caregivers worldwide. Dooher and Byrt (2012) identified “participation” as an important characteristic of empowerment and noted the importance of recognizing how people are treated in society to promote empowerment. Therefore, family caregivers of PWDs must understand issues that they and PWDs face in society in terms of social participation and community involvement.

The category “understanding the social aspects of dementia care” may be specific to dementia, especially in light of global social issues such as stigma. For instance, because of social norms in Japan, caregiving is considered a family role, resulting in a strong tendency to fulfill this responsibility (Kumagai & Ishii-Kuntz, 2016) and to provide care, even under the caregiver's own initiative (Sakakibara et al., 2015). Thus, social attitudes, including the attitudes of family caregivers in Japan, may elicit interest in social and community issues and help promote understanding and appropriate behaviors. Interestingly, this category has not been reported in previous studies (e.g., Che et al., 2006). Therefore, “understanding the social aspects of dementia care” may be specific to the empowerment of Japanese family caregivers of PWDs.

Dooher and Byrt (2012) proposed good communication and relationships between specialists, care recipients, and family caregivers as one dimension of empowerment that specifically promotes care skills and behaviors (e.g., by changing services and policies). The results of this study support that a good relationship between family caregivers of PWDs and the people around them may promote the following: (a) the proactive aspects of dementia care that were acquired through the caregiving experience, (b) creating a relationship that respects PWDs, and (c) understanding of the social aspects of dementia care. Therefore, Category (c) in this study, “building relationships based on mutual understanding of one's surroundings,” was supported as a core category of empowerment for family caregivers of PWDs and may have a positive effect on other categories.

Empowerment includes cognitive, emotional, and behavioral aspects (Dooher & Byrt, 2012). Although many studies have focused on individual psychological empowerment, few have been designed to measure behavioral empowerment in healthcare users (Cyril et al., 2015). The four components identified in this study, covering cognitive, emotional, and behavioral aspects, may represent the characteristics of empowerment among family caregivers of PWDs.

### Implications

The structural components of empowerment presented in this study offer a useful perspective for health professionals in Japan on related assessments and interventions. This perspective may also be applicable to other East Asian countries that share similar aging and dementia issues and family/cultural values. Good relationships between family caregivers of PWDs and the people around them positively affect the other components of empowerment and thus is an issue that deserves greater focus in practice. Furthermore, support for caregivers may be provided based on an assessment of empowerment. Healthcare professionals may develop intervention programs to provide support at different levels of empowerment. For example, family caregivers with low levels of empowerment typically experience anxiety but have limited relationships with health professionals. Thus, health professionals may proactively support family caregivers by providing specific advice on strategies for relieving related anxiety. For family caregivers of PWDs in maturing relationships with those around them, continuous support is needed to help them build good relationships. Finally, the findings have important implications for facilitating research in dementia care from a cultural perspective. However, further study is needed to construct and verify intervention programs for caregivers that are tailored to their level of empowerment.

### Study Limitations

The results of this study may have been affected by sampling bias, as participants were limited to empowered family caregivers in four self-help groups. Although participants were purposively sampled, some perspectives may not have been represented in the sample. Further studies should clarify the nature of empowerment in family caregivers by considering a variety of organizations and community characteristics.

### Conclusions

The findings of this study clarified the components of empowerment among family caregivers of community-dwelling PWDs in Japan and contribute to building a more thorough understanding of empowerment among family caregivers of PWDs in Japan and, potentially, in other East Asian countries. Furthermore, the findings provide a cultural perspective for developing better assessments and interventions for caregivers of PWDs.
